# LIMPRINT: Estimation of the Prevalence of Lymphoedema/Chronic Oedema in Acute Hospital in In-Patients

**DOI:** 10.1089/lrb.2019.0024

**Published:** 2019-04-22

**Authors:** Isabelle Quéré, Sylvie Palmier, Susan Nøerregaard, Jenica Pastor, Martina Sykorova, Eleanor Dring, Peter J. Franks, Susie Murray, Vaughan Keeley, Susan Bermark, Tonny Karlsmark, Norah Kyne, Mary-Paula Colgan, Marie-Michelle Coulombe, Sandrine Mestre, Gregoire Mercier, Christine J. Moffatt

**Affiliations:** ^1^ Department of Vascular Medicine, EA2992, University of Montpellier, CHU Montpellier, Montpellier, France.; ^2^ Department of Dermatology, University Hospital of Montpellier, Montpellier, France.; ^3^ Copenhagen Wound Healing and Lymphoedema Centre, Bispebjerg University Hospital, Copenhagen, Denmark.; ^4^ Épidémiologiste Unité de Recherche Médico-Economique, DIM, CHU de Montpellier, Montpellier, France.; ^5^ School of Health Sciences, University of Nottingham, Queens Medical Centre, Nottingham, United Kingdom.; ^6^ Nottingham University Business School, University of Nottingham, Nottingham, United Kingdom.; ^7^ Centre for Research and Implementation of Clinical Practice, London, United Kingdom.; ^8^ Lymphoedema Service, Royal Derby Hospital, Derby, United Kingdom.; ^9^ Department of Physiotherapy, University Hospital Galway, Galway, Ireland.; ^10^ Department of Vascular and Endovascular Surgery, St. James's Hospital, Dublin, Ireland.; ^11^ Department of Physiotherapy, Calvary Public Hospital Bruce, Canberra, Australia.; ^12^ School of Social Sciences, Nottingham Trent University, Nottingham, United Kingdom.

**Keywords:** lymphedema, lymphoedema, chronic edema, wound, hospital, prevalence, LIMPRINT

## Abstract

***Background:*** To estimate the prevalence of lymphedema/chronic edema (CO) and wounds in acute hospital inpatients in five different countries.

***Methods and Results:*** A point-prevalence study was carried out during working day periods in six general hospitals in four countries (Denmark, France, United Kingdom, and Australia) and one hospital oncology inpatient unit in one other country (Ireland). The study used validated clinical tools for the assessment and collection of data. Data were collected by expert clinicians through interviews and physical examination of the patients present in the wards. A total of 1905 patients could be included and investigated among the 3041 total bed occupancy in the seven hospitals. Lymphedema/CO was present in 723 of them (38%). Main risk factors associated with CO were age, morbid obesity, and heart failure, as well as chair bound immobility and neurological deficiency. History of cellulitis was frequent in patients with CO and wounds (24.8%) and CO alone (14.1%) compared to the 1.5% prevalence in patients without CO.

***Conclusion:*** Lymphedema/CO is very frequent in patients hospitalized in hospital acute wards. It is strongly associated with obesity, venous insufficiency, and heart failure. Our results strongly suggest a hidden health care burden and cost linked to CO independently of chronic wounds.

## Background

Epidemiology studies in lymphedema have frequently focused on single patient populations such as those with cancer rather than on assessing the heterogeneous population that may suffer with the condition.^1^ A public health definition of chronic edema (CO) has been developed and used over the last decades and this reflects the complex pattern of patients who may present with the problem.^2^ The definition defined below has been adopted in the LIMPRINT study:

“Chronic edema (CO) is a broad term used to describe edema that has been present for more than three months and involves one or more of the following areas: limbs, hands/feet, upper body (breast/chest wall, shoulder, and back), lower body (buttocks and abdomen), genital (scrotum, penis, and vulva), head, neck, or face.”

The term of CO includes those with both primary and secondary forms of lymphedema, in addition to venous edema and other factors such as immobility, advanced cancer, obesity, and cardiac and respiratory insufficiency. Within inpatient services, many patients are elderly and will suffer from multiple comorbidities, for which they are receiving many different medications that may also promote CO.

The general population is rapidly aging with increasing comorbidities such as diabetes, immobility, and obesity, which will further challenge health services in all countries of the world.^3^ This will lead to increasing numbers of hospitalized patients who are defined as frail with reduced life expectancy. The concept that CO may be a predictor of frailty as well as being a risk factor for complications such as cellulitis and wounds is worthy of further investigation.

Defining moderate and severe frailty is particularly challenging due to lack of agreed definitions.^4^ Recent National Institute for Health Care and Excellence (NICE) guidance on multiple morbidities has highlighted the need to develop new robust equations to identify patients in primary care or in hospital with reduced life expectancy so that relevant assessments and interventions can be targeted appropriately. Existing equations to predict risk of death are based on biased samples, are insufficiently powered, fail to handle missing data appropriately, are poorly reported, or have poor performance to the extent that NICE has been unable to make a positive recommendation on any tool. The availability of such tools would allow for the identification of patients at risk of unplanned hospital admissions or impending death.

Although recent guidance from NICE on multiple morbidities has recommended tools to predict risk of unplanned hospital admissions, they were unable to identify any equation to reliably predict all-cause mortality. A recent review by NICE of 41 studies to predict all-cause mortality found many methodological limitations, including the omission of key determinants of death, such as age and sex. In addition, the sample sizes were frequently small and unrepresentative of the population.^5^

Lymphedema management is frequently undertaken in an outpatient setting, except for dedicated specialist centers that choose to admit people for intensive periods of treatment (CDT). There is little awareness of how many patients may have CO within inpatient services. It is likely that CO will not be the primary reason for admission and that swelling may be largely ignored as purely a symptom of a related medical condition such as cardiac, renal disease, or deep vein thrombosis.

The methodology developed within the LIMPRINT international epidemiology included methods to be able to screen large numbers of patients within a hospital setting.^6^ Hospital inpatient studies are urgently required to determine the number of people with CO and its impact on health services. There is increasing awareness that CO is associated with major complications such as cellulitis, which have associated mortality as well as being a drain on health service resources.

Cross-sectional based prevalence studies can be undertaken in defined health care settings such as hospitals or care homes where the population is fixed over a short time period. In these settings, a visiting team of researchers can determine the number of people and clinically assess them, thereby deriving an accurate estimation of the prevalence in that setting at a given time point.

Aim: To estimate a point prevalence of CO and wounds as previously defined in inpatients admitted in hospital in five different countries within the LIMPRINT International Project.

### Methodology

The acronym LIMPRINT stands for Lymphoedema IMpact and PRevalence- INTernational. The overall aim was to determine, using a common methodology, the impact and prevalence of CO within health services at a national and international level. This study was undertaken as part of this international project. Specific methodological issues required to undertake this study are defined below.

### Patient inclusion and exclusion criteria

The study patient inclusion criterion was swelling for longer than 3 months (CO), with explicit consent for their data to be transferred into an international database. Patients met exclusion criteria if they were unwilling or unable to participate for whatever reason or were receiving end-of-life care.

## Methods

Sampling frameworks were developed for all participating inpatient hospitals within the study. The screening was undertaken in a single day or over a number of days in larger facilities. Trained staff screened all patients who consented to participate, irrespective of their underlying disease or treatment regimen. The presence and chronicity of edema were confirmed by two methods before CO was judged to be present (a case).

*Confirmation of CO* was based on the following two factors: first, the existence of edema was determined using the “Pitting Edema Test,” which is widely used in clinical practice and been shown to be valid and reliable.^7^ Presence of edema was tested in all body parts, using a standard protocol, including the upper and lower limbs, trunk, face, and neck. Second, edema was judged to be chronic if it had been present for 3 months or more.

### Classification of Primary and Secondary Lymphedema

Following the identification of all cases, a further classification was made of whether the CO was a primary or secondary lymphedema by specialist lymphologists. In those with a secondary lymphedema, additional suspected factors were checked, including venous disease and obesity.

### Other data collection

A body map was used to record the sites and causes of all concurrent wounds. Lower and upper mobility status was defined and information about the history of cellulitis and treatment were also recorded.

### Quality systems

A number of quality systems were used to ensure the accuracy of data capture. The bed capacity of each ward or unit was recorded, plus the number of beds occupied, the number recruited, and the number excluded from consideration and the reasons for this. This enabled the prevalence to be calculated accurately in inpatient hospital settings. Double counting of patients who may have moved within the hospital and be recruited twice was prevented by the allocation of a patient-specific nonidentifiable number. Within each facility, quality monitors were established to check completeness of data from each clinical area before the research teams left the area and all forms were checked by a central coordinator.

### Data analysis

All information was entered into a bespoke database system, which was then downloaded to the statistical packages being used (SAS, Stata 12.0). Data quality checks were used to check the internal consistency of the data. The determination of prevalence was undertaken by dividing the case positives by the total number of patients who had been assessed. These were presented as percentages. Comparison of risk factors between groups was undertaken using Chi-squared analysis, with the results presented as *p* values.

## Results

The LIMPRINT study was undertaken within seven acute health care facilities in five countries. The different hospitals participating in the study were two teaching hospitals in Nottingham (Queen's Medical Centre [QMC]), Nottingham City Hospital (City), United Kingdom; four teaching hospitals Saint Eloi, Gui de Chauliac, Lapeyronie, and Arnaud de Villeneuve in Montpellier, France; two hospitals in Copenhagen, Denmark (Bispebjerg and Frederiksberg Hospital); one general hospital in Canberra, Australia (Calvary Public Hospital Bruce); and one inpatient oncology ward in Galway, Ireland (University Hospital Galway).

### Proportion of patients in hospital within the whole population of the study

**FIG. 1.**
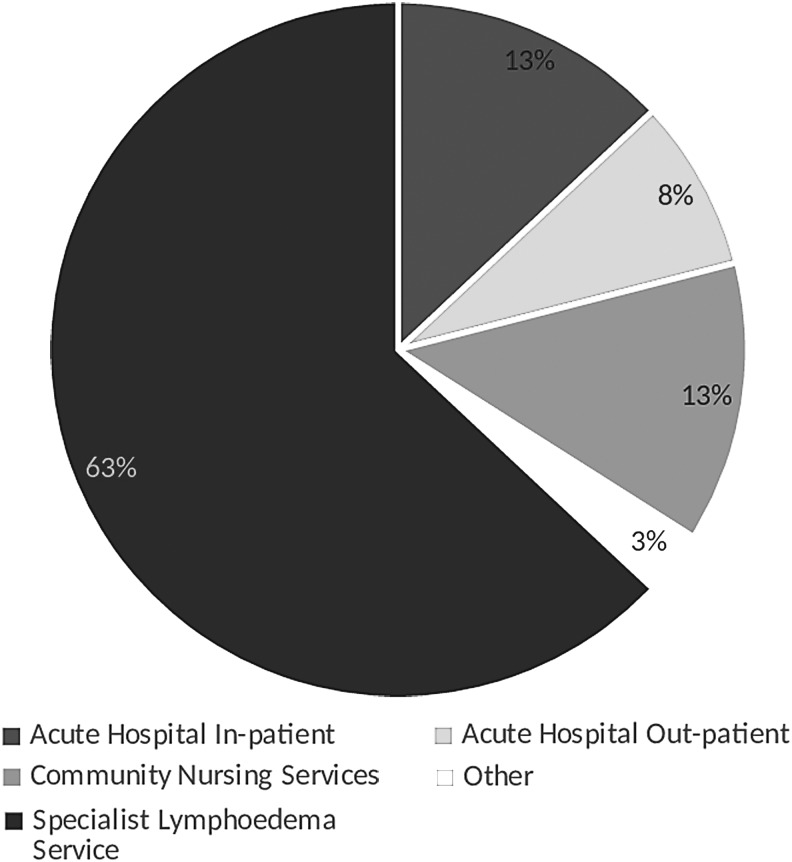
Proportion (%) of patients included from the different settings of care in the nine participating countries of the LIMPRINT Study.

A total of 1905 patients were included and assessed for CO and wounds from a total of 3041 eligible hospital occupants. In two of the hospitals patients who were assessed and found not to have CO were not interviewed further. This made the total number of patients interviewed as 1630. The main reason for exclusion was the absence of informed consent due to cognitive impairment or because the patients were not in their bedrooms at the time of the census. The participants recruited in this study represent 13% of the whole LIMPRINT population (Fig. 1). The prevalence of CO among the patients who had been included was 723/1905 (38%) (Table 1).

**Table 1.** Point Prevalence of Chronic Edema in the Seven Acute Hospital Inpatient Facilities

*Facility*	*Patients with CO (*n*)*	*Patients clinically assessed in wards (*n*)*	*Prevalence of CO in patients clinically assessed (%)*	*Total number of beds in hospital (*n*)*
Montpellier, F	215	726	29.6	1150
Canberra, AU	31	113	27.4	113
Ireland	5	45	11.1	62
QMC, UK	155	324	47.8	634
City Hospital, UK	140	245	57.1	490
Bispebjerg Hospital, DK	134	326^a^	41.1	437
Frederiksberg Hospital, DK	43	126^a^	34.1	141
Total	723	1905	38.0	3041

Only patients under the Oncology Service (45/62 patients) were assessed for swelling at the University Hospital Galway (total bed capacity 708).

^a^ Patients were assessed in these two hospitals.

CO, chronic edema.

### Characteristics of the patients with CO

Mean (SD) age of the patients with CO was 73.0 (15.3) years and 53.9% were female. CO was secondary in 705 patients (97.5%) with a few patients (18, 2.5%) classified as primary lymphedema. CO was recognized as lymphedema associated with cancer in only 57 patients (8.1%). Of the 38 patients with cancer-related swelling, 22 (57.9%) were cancer treatment-related lymphedema and 17 (44.7%) were at a metastatic stage of the disease.

CO was diagnosed in the lower limbs in 690/716 patients (96.4%), mainly below the knee, upper limbs in 102 patients (14.3%), head and neck in 12 patients (1.7%), genitals in 6 patients (0.8%) (1 woman and 5 men), midline, including head, neck, and genitals, in 47 patients (6.6%), 7 (1.0%) were localized on the shoulder and upper chest, abdomen in 26/716 patients (3.6%), and buttocks in 22/716 (3.1%) patients.

A total of 52 patients were morbidly obese (7.4%), 213 were obese (30.2%), and 61 (8.7%) were underweight. Mobility was reduced in most patients being either chair or bed bound in 89 (12.4%) and 90 (12.5%), respectively. Only 228 (31.7%) patients were walking unaided. Comorbidities such as diabetes were seen in 184 patients, a known diagnosis of heart failure in 259 patients, peripheral arterial disease in 60 patients, and neurological deficiency in 124 patients, with 292 patients not suffering any of these comorbidities (Table 2). Aside from cancer, CO was classified according to whether the edema was considered to be caused by other factors. Of the 644 patients with noncancer, secondary edema venous disease was a contributory cause in 262 patients (40.8%), immobility in 337 patients (52.5%), obesity in 100 patients (15.6%), and other causes in 222 patients (34.6%).

**Table 2.** Prevalence of Risk Factors Associated with Lymphedema/Chronic Edema in Hospital Inpatients with Chronic Edema With and Without Wounds Compared to Those Without

	*Total population (*n* = 1630)*	*CO only (*n* = 427)*	*CO and wound (*n* = 296)*	*None (*n* = 907)*	p *(χ^2^)*
Obesity (*n* = 1294)					
Morbidly obese	75	25 (6.1)	27 (9.2)	23 (3.9)	
Obese	351	108 (26.2)	105 (35.8)	138 (23.4)	<0.001
Normal weight	750	245 (59.5)	134 (45.7)	371 (63.0)	
Under weight	118	34 (8.3)	27 (9.3)	57 (9.7)	
Lower limb mobility (*n* = 1287)					
Bed bound	151	47 (11.0)	42 (14.3)	62 (10.9)	
Chair bound	133	56 (13.1)	34 (11.6)	43 (7.6)	<0.001
Walks with aid	437	177 (41.5)	136 (46.4)	124 (21.9)	
Walks unaided	566	147 (34.4)	81 (27.7)	338 (59.6)	
Comorbidities^a^ (*n* = 1630)					
Diabetes Mellitus	291	95 (22.3)	89 (30.1)	107 (11.8)	<0.001
Heart Failure	352	148 (34.7)	111 (37.5)	93 (10.3)	<0.001
Neurological Deficiency	173	77 (18.0)	47 (15.9)	49 (5.4)	<0.001
Peripheral arterial disease	98	24 (5.6)	36 (12.2)	38 (4.2)	<0.001

^a^ Not all comorbidities listed.

The duration of CO ranged between less than 6 months in 179 patients (25.4%) to 144 patients (20.45%) for more than 10 years. A history of cellulitis was present in 131 patients (18.6%) and an episode of any infection was reported in the previous year in 72 patients (10.2%). Recurrent episodes were reported once in 44/67 patients (65.7%), where this was recorded, and at least twice in 23 patients (34.3%). Episodes of cellulitis were responsible for hospitalization in the past year in 49/72 patients (68.1%).

### Treatment of CO

In total, 698/723 patients diagnosed with CO provided information on treatments used. Of these, 260 patients (37.2%) used at least one component of decongestive therapy with the remaining 430 having no treatment. The most frequent were compression garments in 109 patients (15.6%), skin care advice in 49 patients (7.0%), multilayer bandaging in 27 patients (3.9%), wound dressing in the 45 (6.4%) patients with CO and wounds (6.2%), and antibiotics in 22 patients (3.2%). Massage (9), physiotherapy (16), exercise advice (14), pneumatic compression (3), cellulitis advice (6), and psychological support (3) were not frequently reported by patients nor recorded in the medical records. Professionals judged the CO was considered controlled by the team of investigators in 169/718 (23.5%) of the patients and uncontrolled in 490/718 (68.2%) patients. There were a further 59 patients in whom the control was unsure.

### Prevalence of CO and wounds in hospital in-patients (whole population)

Wounds were associated with CO in 296/723 (40.9%) of the patients. The main site of the wound was the lower limb. As wounds are a recognized source of hospitalization and are associated with some COs, data were analyzed as prespecified in the study according to the presence of CO whether associated or not with wounds.

The mean (SD) age was 73.8 (14.4) years in the 427 patients with CO alone, 72.0 (16.4) in the 292 patients with both, and 63.6 (19.1) years in the remaining 541 patients with no wound and no CO (*p* < 0.001). Gender was not different among the three groups. Patients with CO with or without wounds were more frequently morbidly obese compared to patients without CO or wounds, more frequently having mobility problems (65.6% with CO alone, 72.4% CO with wound, and 32.5% with no CO or wound). Diabetes was three times more likely in the group with edema present, four times more likely with heart disease, and five times more likely with neurological deficiency. Patients with CO and wounds differ from those with CO alone only in terms of comorbidities known to be associated with wounds such as diabetes mellitus and peripheral arterial disease.

Finally, patients with CO with and without a wound had a history of cellulitis for 14.1% and 24.8% of them, respectively, when those without had very low rates (1.5%) (*p* < 0.001) (Table 3). In those who were previously diagnosed with cellulitis, cellulitis episodes were frequently responsible for hospitalization in both groups (CO, and CO and wound).

**Table 3.** Prevalence of History of Cellulitis in Patients with Chronic Edema, and Those with Chronic Edema and Wounds

	*CO only,* N *(%)*	*CO and wound,* N *(%)*	*None,* N *(%)*	p *(χ*^2^*)*
History of cellulitis (*n* = 933)				
Yes	58 (14.1)	73 (24.8)	3 (1.5)	<0.001
No	353 (85.5%)	221 (75.2)	225 (98.7)	
Cellulitis within the past 12 months for those with a history of cellulitis (*n* = 131)				
Yes	32 (56.1)	40 (56.3)	1 (33.3)	0.73
No	25 (43.9)	31 (43.7)	2 (66.7)	
Hospitalization for those with a history of cellulitis within past 12 months (*n* = 73)				
Yes	19 (59.4)	30 (75.0)	—	0.29
No	13 (40.6)	10 (25.0)	—	

## Discussion

Lymphedema/CO is a chronic swelling condition that contributes to disability, chronic wounds, and lost quality of life. It is associated with risk factors such as obesity, aging, and cancer treatments, all three increasing at an epidemic level in Europe.^2,8^ The overall impact of lymphedema/CO as a public health problem, however, is underestimated, principally due to the lack of diagnosis and epidemiologic data. These problems pose barriers to optimal management and prevention of the complications of this lifelong condition, resulting in disease progression and hospitalization. This study is the first international study ever performed to assess the prevalence of lymphedema/CO in patients admitted in hospital for any medical or surgical reason as a first step of the identification of the use and cost of health resources allocated to this condition and their complications.

The results indicate that lymphedema/CO is a very prevalent condition with more than 38% (ranging from 11.1 to 57.1%) of the patients showing signs of chronic swelling when they are admitted in hospital, whatever the initial medical or surgical indication for hospitalization. This is in line with the 28.5% prevalence reported in a previous study conducted in the United Kingdom that included the same methodology with a prospective assessment.^9^ The higher rate that we report might be explained by the involvement of secondary and tertiary hospitals, including university hospitals, rather than a regionally based hospital.

Data about the prevalence of chronic wounds are available in the general population and recently estimated at 2.21 per 1000 population.^10^ Data on the prevalence of chronic wounds in different health care settings are also available,^11^ but the focus is mainly on pressure ulcers in hospital and chronic wounds within primary care. The health economic burden that wounds pose to the National Health Service in the United Kingdom has been estimated,^12^ as well as in Germany,^13^ whereas CO has not been recognized to date and therefore lacks robust evidence. It has to be emphasized that management of CO is intrinsically linked to wounds particularly when untreated and this has never been examined before.

The overall prevalence in our study was 38% for the six main hospitals (where all medical and surgical wards had been included) and for the one oncology ward that was included. However, the prevalence was lowest in the oncology ward (11.1%).

In this study CO was found to occur twice more frequently in medical and surgical acute wards than in the oncology ward. However, caution should be taken due to the relatively small sample in the oncology ward population. It could be hypothesized that nutrition and prevention of lymphedema are key components of cancer management that could result in a lower prevalence of CO in the oncology ward than in general medical and surgical wards.^14^

One of the main reasons explaining why CO had never been assessed in inpatient facilities before was the requirement for a physical examination of each patient, which requires a high number of investigators be made available with the relevant clinical skills. CO is also a clinical sign that is not routinely recorded in existing hospital databases. This means that currently, it is impossible to rely on data from health systems to assess the burden and cost of care for CO.

A strength of the methodology was the use of lymphology experts who were responsible for the physical examination of patients and the examination of medical records. The expert investigators were frequently required to support teams of 20–45 general investigators in each hospital. In the United Kingdom, Denmark, and France, the same eight experts undertook the study in each site, ensuring the harmonization and validation of the tools that were used to collect data across the six participating general hospitals. The oncology ward in Ireland used their local lymphology experts to support the study.

The limitations of this study were the complexity of screening patients in busy hospitals. Many were absent from their beds at the time of recruitment as they were undergoing tests or surgical or medical interventions. A number of patients had a cognitive impairment such as dementia which prevented consent being acquired. It is difficult to estimate the bias caused by the exclusion of these patients. However, it is likely to lead to the underreporting of the size of the problem rather than an inflation of the problem. Patients with a cognitive impairment are known to suffer from wounds and poor nutrition, and therefore may have an associated CO. Thus exclusion of these patients will lead to an underestimate of the prevalence.

Such a high number of patients with lymphedema/CO raise the question of whether this is a condition associated with hospitalization or whether it is a preexisting condition occurring before hospitalization, but contributing to the hospital admission. The methodology used cannot answer this question. However, the profile of patients with CO shares some of the characteristics of patients with wounds. They are both 10 years older than those without. A substantial number of risk factors are present in both such as immobility (neurological deficiency, chair bound, and immobility), and significant comorbidities such as heart failure, venous disease, and morbid obesity are shared at the same rate. Contrary to this, diabetes and peripheral arterial disease were found to be more important in patients with wounds compared to those with CO alone. Last, cancer does not appear as the main cause of lymphedema/CO in hospital inpatient settings.

In line with the frailty profile of these patients is the high rate of cellulitis events recorded in patients with lymphedema/CO with and without wounds. Even if logically more prevalent in patients with wounds (24.8%), the rate was 14.1% in patients with lymphedema/CO without wounds, when very low in patients without any CO (1.5%). This means that even if lymphedema is not the main reason for hospitalization, it is associated with significant complications requiring hospitalization such as cellulitis. Moreover, a third of the patients with lymphedema/CO and half of the patients with wounds had a history of hospitalization for cellulitis.

This study shows that over a third of patients in inpatient wards within general hospitals suffer from lymphedema/CO when physically assessed. There is an increasing emphasis on identification of frailty particularly within elderly populations. Results from this study would indicate that CO may prove useful as a marker of frailty. Further research is needed to examine in more depth. At the moment, it remains an open question.

## References

[1] Disipio T, Rye S, Newman B, Hayes S Incidence of unilateral arm lymphoedema after breast cancer: A systematic review and meta-analysis. Lancet Oncol 2013;14:500–515 23540561 10.1016/S1470-2045(13)70076-7

[2] Moffatt CJ, Franks PJ, Doherty DC, Williams AF, Badger C, Jeffs E, Bosanquet N, Mortimer PS Lymphoedema: An underestimated health problem. QJM 2003;96:731–738 14500859 10.1093/qjmed/hcg126

[3] European Commission. The 2012 Ageing Report. 2012 Available at: http://ec.europa.eu/economy_finance/publications/european_economy/2012/pdf/ee-2012-2_en.pdf Accessed on 1214, 2018

[4] Hippisley-Cox J, Coupland C Development and validation of QMortality risk prediction algorithm to estimate short term risk of death and assess frailty: Cohort study. BMJ 2017;358:j4208 28931509 10.1136/bmj.j4208 PMC5606253

[5] Danziger J, Chen K, Cavender S, Lee J, Feng M, Mark RG, Mukamal KJ, Celi LA Admission peripheral edema, central venous pressure, and survival in critically ill patients. Ann Am Thorac Soc 2016;13:705–711 26966784 10.1513/AnnalsATS.201511-737OC PMC5802518

[6] Moffatt CJ, Franks PJ, Keeley V, Murray S, Mercier G, Quere I. The Development and validation of the LIMPRINT methodology. Lymphatic Res Biol (in press) 10.1089/lrb.2018.0081 PMC6636670 30995185

[7] Dai M, Sugama J, Sato A, Tsuchiya S, Sanada H, Moffatt CJ Inter-rater reliability of the AFTD-pitting test among elderly patients in a long-term medical facility. Lymphoedema Res Pract 2015;3:1–7

[8] Keast DH, Despatis M, Allen JO, Brassard A Chronic oedema/lymphoedema: Under-recognised and under-treated. Int Wound J 2015;12:328–333 24618210 10.1111/iwj.12224 PMC7950664

[9] Moffatt CJ, Keeley V, Franks PJ, Rich A, Pinnington LL Chronic oedema: A prevalent health care problem for UK health services. Int Wound J 2017;14:772–781 27917617 10.1111/iwj.12694 PMC7950047

[10] Martinengo L, Olsson M, Bajpai R, Soljak M, Upton Z, Schmidtchen A, Car J, Jarbrink K Prevalence of chronic wounds in the general population: Systematic review and meta-analysis of observational studies. Ann Epidemiol 2019;29:8–15 30497932 10.1016/j.annepidem.2018.10.005

[11] McCosker L, Tulleners R, Cheng Q, Rohmer S, PacellaT, Graves N, Pacella R Chronic wounds in Australia: A systematic review of key epidemiological and clinical parameters. Int Wound J 2019;16:84–95 30259680 10.1111/iwj.12996 PMC7948920

[12] Guest JF, Ayoub N, Mcllwraith T, Ucheqbu I, Gerrish A, Weidlich D, Vowden K, Vowden P Health economic burden that wounds impose on the National Health Service in the UK. BMJ Open 2015;5:e009283 10.1136/bmjopen-2015-009283 PMC4679939 26644123

[13] Heyer K, Herberger K, Protz K, Glaeske G, Augustin M Epidemiology of chronic wounds in Germany: Analysis of statutory health insurance data. Wound Repair Regen 2016;24:434–442 26609788 10.1111/wrr.12387

[14] Roberts S, Chaboyer W, Desbrow B Nutrition care-related practices and factors affecting nutritional intakes in hospital patients at risk of pressure ulcers. J Hum Nutr Diet 2015;28:357–365 24974729 10.1111/jhn.12258

